# Primary Central Nervous System Vasculitis Mimicking Susac Syndrome and Multiple Sclerosis With Long-Term Remission and Spontaneous Resolution of Lesions: A Case Report

**DOI:** 10.7759/cureus.64358

**Published:** 2024-07-11

**Authors:** Akihito Koseki, Youji Suzuki, Shugo Uchida, Naoki Morishita, Yukio Hokazono, Ken Kuriki, Yasuhiro Yamamura, Mari Yoshida, Naoki Sakai

**Affiliations:** 1 Neurology, Yaizu City Hospital, Yaizu, JPN; 2 Diagnostic Pathology, Yaizu City Hospital, Yaizu, JPN; 3 Neurosurgery, Yaizu City Hospital, Yaizu, JPN; 4 Pathology, Aichi Medical University, Nagakute, JPN

**Keywords:** biopsy, spontaneous resolution of lesions, long-term remission, susac syndrome, multiple sclerosis, central nervous system vasculitis

## Abstract

Primary central nervous system vasculitis (PCNSV) is an angiitis localized to the central nervous system (CNS), with various manifestations and no specific biomarkers. Herein, we report a case of PCNSV that presented with an unusual course. A 40-year-old Japanese male developed inner ear symptoms and visual field disturbances. Later, at 42 years of age, the patient developed right hemiparesis and was diagnosed with multiple sclerosis (MS). He received methylprednisolone pulse therapy, which improved his symptoms and resolved most brain lesions. Subsequently, he did not visit the hospital for 13 years, during which time he experienced no relapse. At 55 years of age, he presented to our hospital with fatigue and dizziness. Susac syndrome was suspected because of sensorineural hearing loss and snowball lesions in the corpus callosum. Some of the brain lesions resolved spontaneously. A biopsy was performed on a right frontal lobe lesion, which revealed vasculitis with fibrinoid necrosis, no demyelinating lesions, no amyloid positivity, and no infiltration of atypical lymphocytes. With no evidence of vasculitis in other organs, the patient was diagnosed with PCNSV. The patient was treated with methylprednisolone pulse therapy, followed by oral prednisolone (1 mg/kg/day). The prednisolone was tapered off, and no relapse of symptoms or new lesions on magnetic resonance imaging (MRI) were noted. As observed in this case, even in a scenario suggestive of Susac syndrome or multiple sclerosis, PCNSV should be considered a differential diagnosis and confirmed via brain biopsy.

## Introduction

Primary central nervous system vasculitis (PCNSV) is an angiitis localized to the central nervous system with no known cause [1]. The incidence rate of PCNSV is 2.4 cases per 1,000,000 person-years [2], and the annual relapse rate of PCNSV is 1.4 [3]. With various manifestations and no specific biomarkers, its diagnosis is difficult. Diagnosis is made using angiography or biopsy; a diagnostic error may occur if it is not confirmed by biopsy [1]. No standard treatment for PCNSV has been established, and there is no evidence of the best treatment from randomized controlled trials; it is generally treated with glucocorticoids and immunosuppressive agents [2]. PCNSV with small vessel injury is more likely to relapse than PCNSV with large or medium vessel injury [4,5], and long-term remission without treatment is considered a unique disease course, causing difficulty in diagnosis.

In our practice, we encountered a patient with suspected Susac syndrome and multiple sclerosis (MS) who had a long relapse-free course with spontaneous resolution of some lesions after relapse. Based on brain biopsy findings and the absence of major cerebral artery stenosis in magnetic resonance (MR) angiography, a diagnosis of PCNSV with small vessel injury was made [4]. Herein, we report the details of this case of PCNSV that mimicked Susac syndrome and MS, presenting with an unusual course.

This article was previously presented as a meeting abstract at the 249th Tokai Regional Meeting of the Japanese Society of Internal Medicine on February 19, 2023.

## Case presentation

**Figure 1:**
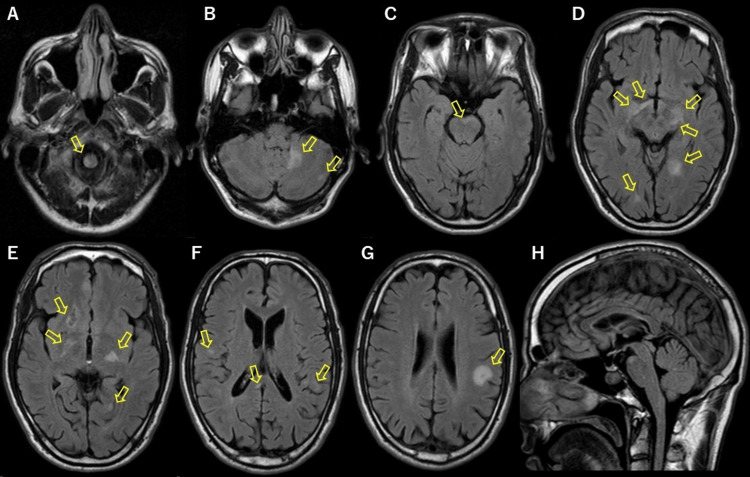
Brain magnetic resonance imaging findings at the time of recurrence at the age of 42 years FLAIR images show hyperintense lesions without contrast enhancement in the right medulla oblongata (A), left cerebellum (B), ventral side of the pons (C), right optic chiasm, bilateral putamen, left cerebral peduncle, subcortical white matter in the bilateral occipital lobe (D), right caudate nucleus, right globus pallidus, left thalamus, subcortical white matter in the left occipital lobe (E), and right splenium of the corpus callosum, subcortical white matter in the right frontal lobe and left parietal lobe (F), and a 20-mm-diameter rounded lesion in the subcortical white matter in the left parietal lobe (G). FLAIR images show no snowball lesions (H). FLAIR: fluid-attenuated inversion recovery

A 40-year-old Japanese male presented with vertigo, nausea, tinnitus, hearing loss in the left ear, left nasolateral visual field defect, and occipital headache without specific triggers. The patient had a history of atopic dermatitis and bronchial asthma but no family history of inherited neurological diseases. Cerebrospinal fluid (CSF) showed pleocytosis with 31 cells/µL (98% mononuclear cells) and a protein level of 89 mg/dL. Furthermore, the CSF was positive for oligoclonal bands (OCBs); however, data on the number of bands was lost. No bands were observed in the serum. Blood anti-aquaporin-4 antibody was not detected. MR imaging (MRI) showed multiple T2 hyperintense lesions in the dorsal C3/4 cervical spinal cord and cerebral white matter with contrast enhancement of the cervical spinal cord lesions and some cerebral lesions (images were lost). The patient was diagnosed with MS using the 2005 revision of the McDonald Criteria, which were the latest diagnostic criteria at the time [6]; however, he was considered to have MS with atypical findings owing to the presence of a headache. His symptoms improved without immunotherapy. The patient subsequently developed mild right upper and lower extremity paralysis, without any specific triggers, at 42 years of age. The patient did not receive any disease-modifying treatment for MS between the ages of 40 and 42 years. His right upper extremity muscle strength was 5/5 on manual muscle testing (MMT) but was positive for the digiti quinti sign. His right lower extremity muscle strength was 4/5 on MMT. CSF analysis showed pleocytosis with 50 cells/µL (89% mononuclear cells), a protein level of 95 mg/dL, and positive oligoclonal bands. Fluid-attenuated inversion recovery (FLAIR) images showed hyperintense lesions without contrast enhancement in the right medulla oblongata, left cerebellum, ventral side of the pons, right optic chiasm, bilateral putamen, left cerebral peduncle, right caudate nucleus, right globus pallidus, left thalamus, and right splenium of the corpus callosum; subcortical white matter in the bilateral occipital, right frontal, and left parietal lobes; and a 20-mm-diameter rounded lesion in the subcortical white matter in the left parietal lobe. FLAIR images showed no snowball lesions (Figure 1).

The patient was diagnosed with MS [6] and received methylprednisolone pulse therapy. His symptoms improved, and all hyperintense lesions on FLAIR images, except for the lesions in the right caudate nucleus and the left cerebral peduncle, had resolved. After his symptoms improved, the patient stopped visiting the hospital for 13 years, during which he did not experience relapse. We have no record of any symptoms; however, we cannot confirm their absence. The patient denied any sensations of continuing fatigue, dizziness, limb movement difficulty, visual impairment, or hearing difficulties during this time. Furthermore, he was able to perform his job and drive without any issues. No concerns were raised by family members or colleagues at his workplace regarding any abnormal symptoms. The patient was subsequently admitted to our hospital at 55 years of age on day X for continuous malaise and dizziness for three months without any specific trigger.

On admission, the patient's body temperature was 36.8℃, heart rate was 100 beats/minute, and blood pressure was 111/79 mmHg. His respiratory rate was 12 breaths/minute, with an oxygen saturation of 97% on room air. His height and weight were 172 cm and 65 kg, respectively. The patient was alert and interactive with no headache. An atopic dermatitis rash was present; however, no other rashes were observed, nor was edema of the extremities. Unilateral spatial neglect was not present. The diameter of the pupils was 3 mm bilaterally, with normal light reflexes, no nystagmus, normal eye movements, and no impairment of other parts of the cranial nervous system. Muscle strength was normal, deep tendon reflexes were mildly increased in the extremities but did not differ bilaterally, and pathological reflexes were absent. No abnormalities were observed in the sensory system, motor coordination, or autonomic nervous system. Gait was normal, as were the results of the remaining physical examinations.

**Figure 2:**
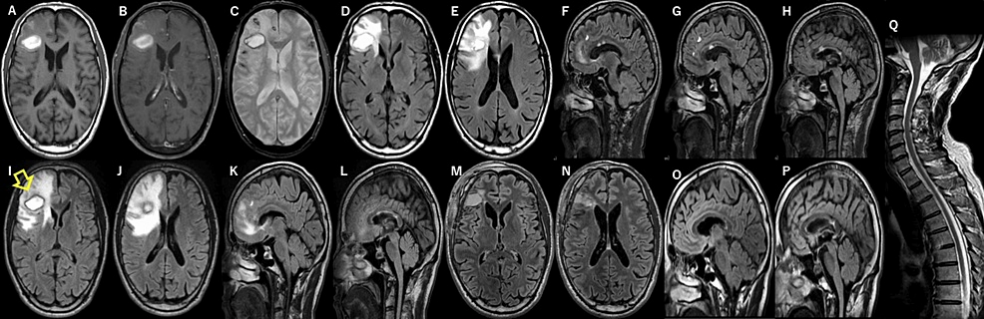
Brain and spinal cord magnetic resonance imaging findings on admission at the age of 55 years and before and after brain biopsy T1-weighted image shows a hyperintense lesion in the right frontal lobe (A). Contrast enhancement is not observed in this lesion (B). T2* image shows a hyperintense lesion with scattered microhemorrhagic changes in the bilateral cortical and subcortical areas (C). FLAIR images show edematous lesions around the right frontal lobe lesion (D and E) and snowball lesions in the corpus callosum (F-H). On day X+21 before brain biopsy, FLAIR images show no change in the T1 and T2 hyperintense lesions in the right frontal lobe, but the surrounding edematous lesions are enlarged (I and J). We performed a brain biopsy of the lesion in the right frontal lobe (I) (arrow). Some snowball lesions in the corpus callosum have resolved, and some have decreased in size (K and L). On day X+76 after brain biopsy and before treatment, the edematous lesions around the right frontal lobe lesion are reduced on FLAIR images (M and N). The residual snowball lesions have resolved (O and P). Spinal cord magnetic resonance imaging showed no lesions (Q). FLAIR: fluid-attenuated inversion recovery

Blood tests revealed no abnormalities in blood counts or coagulation tests, along with a normal C-reactive protein level and erythrocyte sedimentation rate. No anti-thyroid, anti-nuclear, anti-DNA, anti-SS-A, anti-SS-B, anti-neutrophil cytoplasmic, anti-aquaporin-4 (cell-based assay), or anti-myelin oligodendrocyte glycoprotein (cell-based assay) antibodies were detected. Antigen and antibody test results for syphilis, hepatitis B virus, hepatitis C virus, human immunodeficiency virus, and human T-cell leukemia virus type 1 infections were negative, and urinalysis results were normal. Cerebrospinal fluid showed an initial pressure of 90 mmH₂O, pleocytosis with 33 cells/μL (89% mononuclear cells), a protein level of 89 mg/dL, glucose level of 48 mg/dL, immunoglobulin G index of 0.71, and positive oligoclonal bands; no bands were observed in the serum. No *Cryptococcus* or *Aspergillus* antigens were detected. Polymerase chain reaction tests for herpes simplex virus and varicella zoster virus yielded negative results. The results of the CSF bacterial cultures were negative; cerebrospinal fluid cytology revealed no malignant cells. The corrected visual acuity was 20/20 in both eyes, visual field and fundus examination results were normal, and fluorescein angiography showed no hyperfluorescence or vascular obstruction in the vessel wall. An audiogram revealed mild sensorineural hearing loss on the right side. Cognitive function tests revealed a Mini-Mental State Examination score of 29/30 and a Frontal Assessment Battery score of 17/18. MRI of the head performed on day X+2 showed a 30-mm-diameter lesion with edematous lesions in the right frontal lobe, which were T1 and T2 hyperintense lesions without contrast enhancement. Four small lesions with the same intensities as the 30-mm lesion were located anterior and superior to this lesion. Additionally, snowball lesions in the corpus callosum and T2 and FLAIR hyperintense lesions in the white matter of the right occipital lobe and ventral side of the right cerebellar hemisphere were noted. T2^*^-weighted images revealed scattered microhemorrhagic changes in the subcortex (Figure 2). MRI of the spinal cord showed no lesions (Figure 2). MR angiography showed no major cerebral artery stenosis. Digital subtraction angiography and computed tomography angiography were not performed because the patient had bronchial asthma. Endoscopic examination demonstrated no evidence of vasculitis or malignancy, and whole-body positron emission tomography-computed tomography revealed no abnormal findings.

On day X+21, MRI showed that some of the snowball lesions in the corpus callosum and the right cerebellar lesion had resolved, whereas some of the remaining snowball lesions had decreased in size. The T1 and T2 hyperintense lesions in the right frontal lobe remained unchanged. However, the surrounding edematous lesions were enlarged (Figure 2). During a detailed examination, the patient's malaise and dizziness were mild without treatment and resolved spontaneously, with no symptoms observed after day X+21.

**Figure 3:**
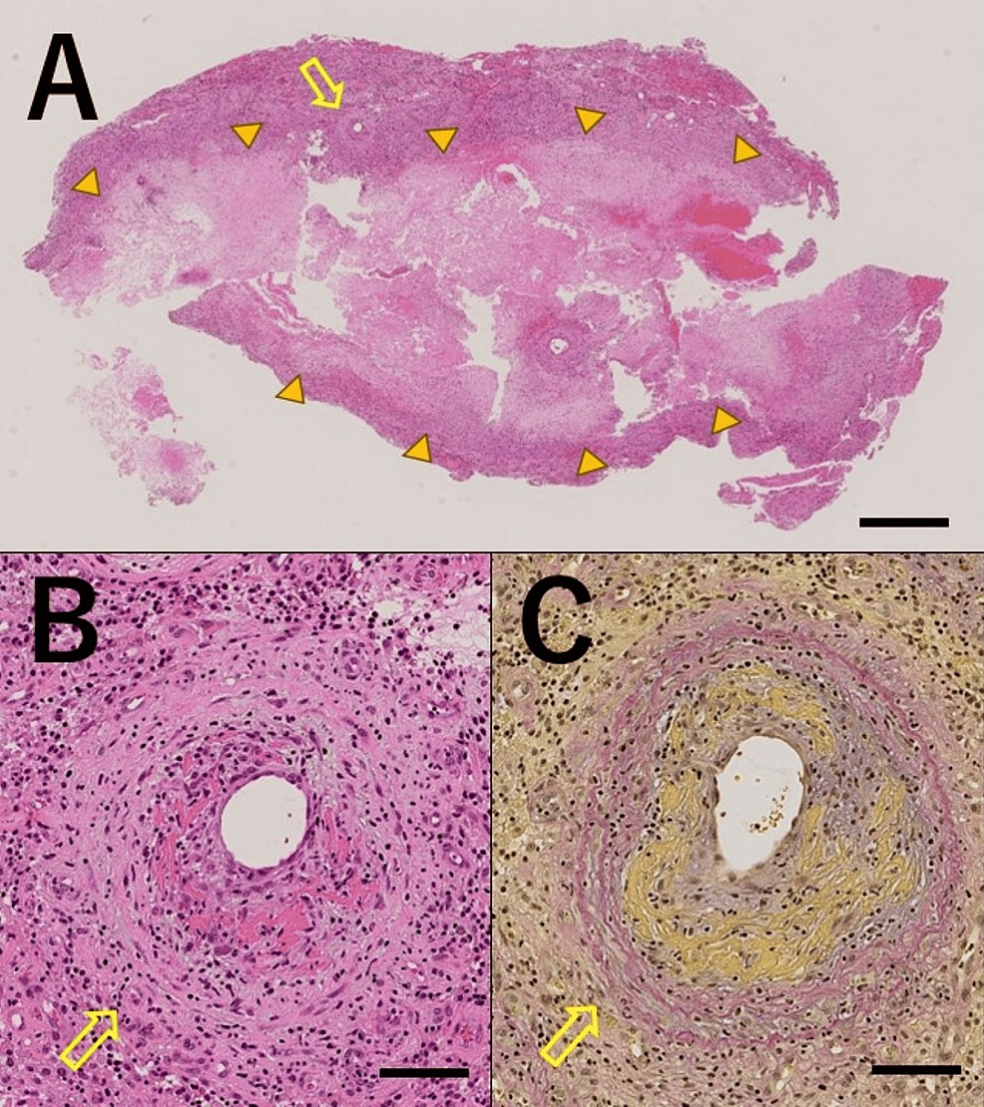
Histopathological findings of brain biopsy Hematoxylin and eosin staining shows a hemorrhagic necrotic lesion surrounded by granulation including small vessels with fibrinoid necrosis (scale bar = 1 mm) (A). Hematoxylin and eosin staining (B) and Elastica van Gieson staining (C) show small vessels with fibrinoid necrosis scattered throughout the granulation tissue (scale bar = 100 μm). Arrow: small vessel with fibrinoid necrosis, arrowhead: hemorrhagic necrotic lesion

On day X+23, a biopsy was performed on a lesion in the right frontal lobe (Figure 2). The specimen mostly comprised hemorrhagic necrotic nests surrounded by granulation tissue with hemosiderin deposits. Small vessels with fibrinoid necrosis were scattered throughout the granulation tissue at the margin of the necrotic nests. No demyelinating lesions were observed, and there were no positive deposits for Congo Red staining or direct fast scarlet staining and no positive images of immunostaining for amyloid beta or AT8. There was no infiltration of atypical lymphocytes suggestive of malignant lymphoma (Figure 3). Based on pathological findings, the patient was diagnosed with vasculitis with fibrinoid necrosis. With no evidence of vasculitis in other organs, we ultimately diagnosed the patient with PCNSV according to the diagnostic criteria described by Calabrese and Mallek [7] and Rice and Scolding [8].

MRI performed on day X+76 (pretreatment) revealed that the edematous lesions around the right frontal lobe lesion, which had enlarged before the brain biopsy, had decreased in size, and the remaining snowball lesions had resolved (Figure 2). The patient was treated with methylprednisolone pulse therapy, followed by oral prednisolone (1 mg/kg/day). The prednisolone was tapered off to 5 mg in combination with oral azathioprine (50 mg/day). Although some T2 hyperintense lesions in the cerebrum remained, there was no relapse of symptoms, and no new lesions have been found on MR images since day X+76.

## Discussion

PCNSV affects blood vessels of various sizes, presents with various symptoms, and lacks specific biomarkers. PCNSV must be differentiated from many other diseases (Table 1) [1]. The diagnostic criteria used herein included those by Calabrese and Mallek [7] and Rice and Scolding [8], and this case met both these criteria. This case met all three of the following diagnostic parameters proposed by Calabrese and Mallek [7]: (1) the presence of an unexplained neurological deficit after thorough clinical and laboratory evaluation, (2) documentation by cerebral angiography and/or tissue examination of an arteritic process within the central nervous system, and (3) no evidence of a systemic vasculitide or any other condition to which the angiographic or pathologic features could be secondary. This case met the following diagnostic criteria proposed by Rice and Scolding [8] and was classified as "definite." The patient presented with suspected CNS vasculitis. Neither primary systemic vasculitic syndrome nor other possible etiologies were noted. Biopsy results revealed positive CNS histology, specifically CNS angiitis with vessel wall damage present [8]. Histologically, PCNSV is classified into three types: granulomatous, lymphocytic, and necrotizing [9]. Cohort studies have shown that the characteristics and prognosis of PCNSV vary according to histological classification; for example, the lymphocytic type has a better prognosis than the granulomatous or necrotizing type [10]. Our patient was diagnosed with the necrotizing type, as fibrinoid necrosis was detected in the vessel wall [9].

**Table 1:** Mimics of PCNSV PCNSV: primary central nervous system vasculitis, RCVS: reversible cerebral vasoconstriction syndrome, CADASIL: cerebral autosomal dominant arteriopathy with subcortical infarcts and leukoencephalopathy, CARASIL: cerebral autosomal recessive arteriopathy with subcortical infarcts and leukoencephalopathy, CARASAL: cathepsin A-related arteriopathy with strokes and leukoencephalopathy, MELAS: mitochondrial encephalopathy, lactic acidosis, and stroke-like episodes, SARS-CoV-2: severe acute respiratory syndrome coronavirus 2, ADEM: acute disseminated encephalomyelitis, NMOSD: neuromyelitis optica spectrum disorder, MOG: myelin oligodendrocyte glycoprotein, ABRA: amyloid beta-related angiitis, CNS: central nervous system, ANCA: anti-neutrophil cytoplasmic antibody, CLIPPERS: chronic lymphocytic inflammation with pontine perivascular enhancement responsive to steroids Adapted from Kraemer and Berlit (2021) [1]

Mimics of PCNSV (adapted from Kraemer and Berlit (2021) [1])	
Noninflammatory vasculopathies	
	RCVS
	Atherosclerosis
	Neurofibromatosis
	Fibromuscular dysplasia
	CADASIL
	CARASIL, CARASAL
	MELAS
	Col COL4A1 and COL4A2 syndrome
	Sneddon's syndrome
	Divry van Bogaert syndrome
	Moyamoya angiopathy
	Osler's disease
	Hypercoagulable state
Infectious vasculopathies	
	Emboli from subacute bacterial endocarditis
	Basal meningitis caused by tuberculosis or fungal infection
	Bacterial infections (borreliosis, lues)
	Progressive multifocal leukoencephalopathy
	Viral infections (varicella zoster, herpes simplex, SARS-CoV-2)
Demyelinating syndromes	
	Multiple sclerosis
	ADEM
	NMOSD
	Anti-MOG-disease
Metabolic diseases	
	Fabry's disease
ABRA as an inflammatory form of cerebral amyloid angiopathy	
CNS vasculitis as part of a primary systemic vasculitis	
	Large vessel vasculitis
Giant cell arteritis	
Takayasu arteritis	
	Medium vessel vasculitis
Polyarteritis nodosa	
Kawasaki disease	
	Small vessel vasculitis
ANCA-associated vasculitides (e.g., granulomatosis with polyangiitis, eosinophilic granulomatosis with polyangiitis, microscopic polyangiitis)	
Immune complex deposition (e.g., Henoch-Schönlein purpura, cryoglobulinemia)	
Systemic autoimmune and rheumatic diseases	
	Neurosarcoidosis
	Neuro-Behcet's disease
	Rheumatic syndromes (e.g., systemic lupus erythematosus, Sjögren syndrome, scleroderma)
Other autoimmune diseases	
	Susac syndrome
	Autoimmune encephalitis
	CLIPPERS
Malignant diseases	
	Primary CNS lymphoma
	Vascular lymphoma
	Carcinomatous meningitis

While PCNSV has a chronic progressive or relapsing-remitting course, in a cohort study of 191 patients, 41 (21.5%) remained in long-term remission for >12 months after treatment discontinuation [10]. PCNSV with small vessel injuries has been reported to relapse more frequently than PCNSV with large or medium vessel injuries [4,5]. Our patient remained in long-term remission from the time of initial onset until the first relapse at 42 years of age, as well as from the initial relapse until the second relapse at 55 years of age. The patient was considered to have PCNSV with small vessel injury because MR angiography showed no major cerebral artery stenosis. In this type of PCNSV, long-term remission with no treatment is considered a unique disease course. PCNSV is usually treated with therapeutic intervention; therefore, no cohort studies have evaluated the natural history of PCNSV after a long period without treatment from diagnosis. Nevertheless, a previous study analyzing the course of PCNSV with induction therapy without maintenance therapy reported that only 20% of patients achieved a relapse-free course, with a modified Rankin Scale (mRS) ≤ 2 at the last follow-up and a median follow-up of 55 months [11]. Furthermore, a cohort study demonstrated that small vessel vasculitis relapsed more frequently than medium/large vessel vasculitis, with 89% of cases having relapsed at the final follow-up at a median of 1,222 days [12]. Given that our case had a relapse-free course with good outcomes, it was considered more unique, causing difficulty in the diagnosis. However, no imaging studies were performed during the symptom-free period, and we were unable to confirm the presence of any imaging changes during that time. Moreover, the patient may have experienced mild symptoms, with little or no awareness of disease activity.

In this case, we suspected Susac syndrome based on the findings of the physical examination at the time of initial onset and relapse and the snowball lesions in the corpus callosum. Susac syndrome is a microvascular disorder that presents as central nervous system dysfunction, visual disturbances, and sensorineural hearing loss [13]. However, many patients present with only a few of these symptoms [13]. The diagnosis of Susac syndrome is based on the criteria proposed by the European Susac Consortium [14]. In this case, the patient presented with visual field disturbances, sensorineural hearing loss, and central nervous system involvement at the time of initial onset at 40 years of age. However, the MRI images were lost, and fluorescein angiography was not performed; thus, it could not be determined whether the patient met the diagnostic criteria for Susac syndrome. At 55 years of age, the patient presented with dizziness and mild sensorineural hearing loss. MRI revealed snowball lesions in the corpus callosum, which is a feature of Susac syndrome [15]. Based on these findings, the patient met the "possible" Susac syndrome diagnostic criteria. Unlike PCNSV, in which blood vessels of various sizes are injured, Susac syndrome is an immune-mediated disease that affects only microvessels and is characterized by vascular endothelial swelling and microvascular obstruction [13,16]. The histological findings also differ; patients with Susac syndrome show vessel wall thickening and perivascular inflammation only in the microvasculature, such as the meningeal and parenchymal arterioles and capillaries, whereas those with PCNSV show inflammation with vessel wall thickening, vessel wall destruction, and perivascular inflammation in vessels of various sizes [15], possibly due to immune responses in the cerebral artery wall [17]. In the present case, histology demonstrated vasculitis with fibrinoid necrosis; therefore, Susac syndrome was considered unlikely. Although a previous report documented the presence of PCNSV with snowball lesions in the corpus callosum, the included cases differed from ours in that angiography revealed vascular stenosis, which indicates medium vessel injuries [15]. Confirming abnormalities using angiography in patients with PCNSV with small vessel injury is challenging; hence, its presence in a patient may not be reliably identified by angiography.

In this case, some snowball lesions in the corpus callosum and the right cerebellar lesion resolved without treatment, whereas the edematous lesions around the right frontal lobe expanded. There is one previous report in the literature of an untreated case of PCNSV that presented with partial spontaneous resolution and partial lesion enlargement based on imaging findings; these findings have been judged to represent regression and progression of inflammation [18]. Similarly, in the present case, some T2 hyperintense lesions resolved and others expanded without treatment, suggesting that one of the types of PCNSV is a condition in which lesions change throughout the natural course of the disease. The spontaneous resolution of lesions suggests that disease activity could be spontaneously suppressed without treatment; this may have led to long-term remission without treatment in this case. On MR images acquired after the brain biopsy and before medication, the edematous lesions around the right frontal lobe lesion, which had enlarged before the brain biopsy, were reduced. The mechanism of reduction in the edematous lesions without treatment after the removal of the right frontal lobe lesion is unknown; therefore, further case studies are required.

The patient may have had other diseases, such as MS, multiphasic disseminated encephalomyelitis, anti-myelin oligodendrocyte glycoprotein antibody-associated disease, or Susac syndrome, at the time of initial onset and first relapse at the age of 42 years and then separately developed PCNSV at the age of 55 years after the improvement of the other diseases. However, we believe PCNSV persisted in the patient since its onset. This is because all three exacerbations were similarly less symptomatic than the extent of the lesions on images suggested. Additionally, the symptoms and lesions responded favorably to steroid treatment. The cerebrospinal fluid findings were the same for all three recurrences, and in each recurrence, there was a similar mild increase in protein levels and cell count with OCB positivity.

This patient presented with PCNSV characterized by small vessel injuries, typically associated with a high relapse rate. The pathology was of the necrotizing type with a relatively poor prognosis. However, the patient exhibited a unique clinical course with an extended period of remission and spontaneous improvement, which made it difficult to distinguish PCNSV from Susac syndrome and MS.

## Conclusions

In this case, the patient was diagnosed with MS and remained untreated for a long period without relapse. Susac syndrome was clinically suspected. The patient had an unusual history of spontaneous resolution of some lesions, and a brain biopsy confirmed the diagnosis of PCNSV. The findings from the present case indicate that PCNSV may have a unique course or mimic other diseases, such as Susac syndrome and MS, thus requiring a brain biopsy to confirm the diagnosis.
